# Management of Chloroquine and Hydroxychloroquine Poisoning: Do Not Miss the Time of Tracheal Intubation and Mechanical Ventilation

**DOI:** 10.5811/westjem.2020.10.49826

**Published:** 2021-01-11

**Authors:** Bruno Megarbane, Azzurra Schicchi

**Affiliations:** * Paris-Diderot University, Lariboisière Hospital, Department of Medical and Toxicological Critical Care, INSERM UMRS-1144, Paris, France; † IRCCS Maugeri Hospital and University of Pavia, Department of Toxicology, Pavia, Italy

To the Editor:

We would like to comment on Lebin and LeSaint’s overview of chloroquine/hydroxychloroquine (CQ/HoCQ) toxicity and management.^1^ The authors focused on the indications and administration modalities of hypertonic sodium bicarbonate, diazepam, and epinephrine. Surprisingly, they did not consider the role and indications of tracheal intubation and mechanical ventilation, while representing the mainstay of treatment.

Lebin and LeSaint recommended the administration of high-dose diazepam (2 milligrams per kilogram intravenously over 30 minutes) in severely CQ/HoCQ-poisoned patients.^1^ As stated, this recommendation is based on observations from the 1950s by French military doctors in Africa reporting that patients referred with mixed CQ/diazepam overdoses had better outcome than patients referred with CQ exposures alone. Thereafter, experimental and clinical studies investigated the utility of diazepam to treat CQ-poisoned patients. Preliminary in vitro investigations using rat left ventricular papillary muscles exposed to CQ and diazepam suggested that diazepam-attributed protective cardiovascular effects in CQ poisoning cannot be explained by an improvement in the intrinsic cardiac mechanical properties.^2^ Recently, in vivo rat models of CQ toxicity used to assess diazepam, clonazepam and Ro5-4864 administered prior, during and after CQ, and high-dose diazepam eventually co-administered with epinephrine, demonstrated that neither diazepam nor other ligands for benzodiazepine-binding sites were effective to protect against or attenuate CQ-induced cardiotoxicity.^3^ Diazepam-attributed augmentation of co-administered positive inotrope effects was the only effect that contributed to reduce cardiotoxicity.

Similarly, in a double-blind placebo-controlled study, diazepam did not reverse CQ-induced clinical and electrocardiographic effects in moderate intoxication.^4^ Altogether, these findings strongly suggested not administering high-dose diazepam in spontaneously breathing CQ/HoCQ-poisoned patients due to its ineffectiveness and to the elevated risk of aspiration pneumonia.^5^ Clearly and in contrast to what is stated in the review paper, the belief that diazepam may improve CQ/HoCQ-induced-vasodilation or dysrhythmic effects is illusive. Although used in the reference trial,^6^ the main role in the beneficial outcome of CQ/HoCQ-poisoned patients among all administered treatments should go to early tracheal intubation, mechanical ventilation, and epinephrine infusion.

High-dose IV epinephrine infusion should also be used with caution. As stated,^1^ epinephrine is the vasopressor of choice to reverse CQ/HoCQ-induced hypotension, especially since toxicity combines vasodilatation and decreased myocardial contractility. Due to their fast sodium channel-blocking properties, CQ/HoCQ slow intraventricular conduction leading to the development of unidirectional block and re-entrant circuits that may degenerate into monomorphic ventricular tachycardia and fibrillation.^7^ By accelerating the heart rate, epinephrine limits these effects. However, the optimal heart rate to target is unclear. As shown with class-I antiarrhythmic drugs, epinephrine-induced tachycardia may increase CQ/HoCQ binding affinity of sodium-channel receptors that vary in the course of the cardiac cycle and thus enhance the frequency-dependent drug toxicity.^8^ Additionally, elevated doses of epinephrine may be responsible for excessive vasoconstriction, ventricular arrhythmia, lactic acidosis, and myocardium stunning. Thus, preferring norepinephrine/dobutamine combination may be an attractive option, although this alternative has not been evaluated in comparison to epinephrine at the bedside. Managing severely CQ/HoCQ-poisoned patients cannot be limited to blood pressure monitoring but should include accurate hemodynamic parameter measurement in the intensive care unit.

Because of their direct cardiotoxicity through voltage-dependent sodium- and potassium-channel blockade, CQ/HoCQ may be responsible for rapid-onset dysrhythmia.^9^ Hypokalaemia from impairment of outward potassium currents additionally favors polymorphic ventricular reentry dysrhythmias by slowing repolarization and prolonging the effective refractory period. Since CQ/HoCQ-poisoned patients are at risk of vomiting, drowsiness, hyperexcitability with restlessness, seizures, consciousness impairment (although rare), and central respiratory depression, they may develop aspiration pneumonia, atelectasis, and alveolar hypoventilation.^9^ Pulmonary edema from either cardiogenic or non-cardiogenic origin with alveolar hemorrhage may occur. The resulting hypoxemia rapidly worsens cardiotoxicity resulting in sudden cardiac arrest. For all these reasons, early tracheal intubation to secure airways and ventilation has been recommended in severely CQ/HoCQ-poisoned patients, as early as in the prehospital setting, before the onset of complications. Intubation is required if at least one prognostic factor of death is present (ie, presumed ingested dose of ≥4 grams, systolic blood pressure ≤100 millimeters mercury, and QRS complex duration ≥100 milliseconds).^2^ Noteworthy, by contrast to psychotropic drug poisonings, intubation is not guided by the Glasgow Coma Scale score nor by the signs of acute respiratory distress.

In conclusion, due to expected CQ/HoCQ overdoses following growing prescriptions in COVID-19 patients, physicians should keep in mind the importance of early intubation and mechanical ventilation when reading the remarkable Lebin and LeSaint’s brief overview.

## References

[1] Lebin JA, LeSaint KT (2020). Brief review of chloroquine and hydroxychloroquine toxicity and management. West J Emerg Med.

[2] Riou B, Lecarpentier Y, Barriot P (1989). Diazepam does not improve the mechanical performance of rat cardiac papillary muscle exposed to chloroquine in vitro. Intensive Care Med.

[3] Hughes DA (2020). Acute chloroquine poisoning: a comprehensive experimental toxicology assessment of the role of diazepam. Br J Pharmacol.

[4] Clemessy JL, Angel G, Borron SW (1996). Therapeutic trial of diazepam versus placebo in acute chloroquine intoxications of moderate gravity. Intensive Care Med.

[5] Mégarbane B, Goldgran-Tolédano D, Résière D (2001). Pneumopathie bactérienne favorisée par le diazépam lors d’une intoxication aiguë à la chloroquine. Presse Med.

[6] Riou B, Barriot P, Rimailho A, Baud FJ (1988). Treatment of severe chloroquine poisoning. N Engl J Med.

[7] Bruccoleri RE, Burns MM (2016). A literature review of the use of sodium bicarbonate for the treatment of QRS widening. J Med Toxicol.

[8] Weirich J, Antoni H (1998). Rate-dependence of antiarrhythmic and proarrhythmic properties of class I and class III antiarrhythmic drugs. Basic Res Cardiol.

[9] Jaeger A, Sauder P, Kopferschmitt J (1987). Clinical features and management of poisoning due to antimalarial drugs. Med Toxicol Adverse Drug Exp.

